# Observations on the Effects of Nitrous Acid and Opium in the Cure of Dysentery

**Published:** 1800-05

**Authors:** T. Hope

**Affiliations:** Chatham


### To JOHN PEARSON, Efq. Golden Square.

Dear Sir,

I Return you fincere thanks for your indulgent attention to my laft; the beneficial effects of your advice have already been experienced by my patient; and in obedience to your wifh, I have tranfmitted to you, my experience of the good effects of Nitrous Acid and Opium in the cure of Dyfentery. If, on perufal, you think it worthy the infpection of the public, you will do me particular honour by forwarding it to Dr. Bradley, for his Publication. I am,

Dear Sir,

With high efteem,

Your obedient, humble fervant,

*Chatham, March* 28, 1800. T. HOPE.

---

### *Obfervations on the Effects of Nitrous Acid and Opium in the Cure of Dysentery.*

A defire of throwing in my mite to the public treafury, induces me to come forward, however unwilling I otherwife feel, to inform fociety of the benefit derived to many of my patients in dyfentery, by the ufe of the nitrous acid, conjoined with opium. It is perfectly unneceffary for me to attempt to depreciate the effects of almoft all remedies that have been in general recommended; the various lifts of fhips journals, and army reports, too ftrongly prove, that the beft practice, founded on a knowledge of the difeafe, and powers of remedies employed, have been frequently inefficient, and always doubtful.

Writers on this difeafe, have confidered dyfentery as peculiar to hot countries; and when I compare the obfervations of Dr. Lind with thofe of Dr. Trotter, and other able navy furgeons, I am led to infer, and fuggeft my inference for further and more able confideration, whether the fame caufes, acting in a diverfe manner, may not be productive of yellow fever? If fo, whether the remedy, as a general one, or operating by general laws, may not alfo be equally beneficial in both diforders? This, however, is certain, as far as the narrow limits of my practice will warrant me to conclude, that, with refpect to dyfentery, it is both fafe and certain.

I neither afk nor expect, merit to be attached to the difcovery; it was purely accidental; nor would it ever have become public from me, ſave in the ſmall circle of my medical friends, where it has alſo been uſeful, but from a perſuaſion of duty I owe to the profeſſion, and to ſociety; and I earneſtly hope thoſe gentlemen, whoſe ſituations place them in the midſt of this calamity, will favor humanity by giving it a trial; and as I have no doubt of its efficacy, entreat they will forward its uſe to the utmoſt of their power.

From the narrow limits of my paper, my ſelections are but few; and, as I never intended them for public inſpection, not ſo correct as I could wiſh; my ſituation is not where the diſeaſe occurs frequently, which will, it is hoped, ſufficiently apologize for any defects that may appear.

The firſt occaſion of its uſe was remarkable: A young man, of ſobriety and temperance, had ſuffered long with dyſentery, and had been attended by a friend of mine for ſome time, who recommended thoſe remedies that high authority and experience pointed out for relief; but finding no advantage, he ſent for me, expecting more might be done. Nothing however recommended was ſuccesſful; and as I could but go over the old ground, no proſpect of relief appeared; indeed, his death was daily expected. At this time, a woman who lived with him, was attacked with dyſentery, accompanied with extreme thirſt. An acid occurred to my thoughts for relief; but fearing it might produce unpleaſant effects, opium was added thereto; the following drops were ſent: ℞. Acid nit. ʒij. opii gr. ij. aq. puræ ℥ij. M. ft. cap. coch. 1 m. ter 4terve die in vehiculo. The effect produced was ſo great, that my dying patient, unknown to me, begged to partake with her; and when I ſaw him next morning, which, to my great ſurpriſe, was with a cheerful countenance, he told me, that "he hoped if ever I had a patient ill with his complaint, I would never fail to ſend thoſe drops that I had ſent for the woman; they had relieved his pain by the firſt doſe, and he was ſure he ſhould live now, for they had ſaved his life this time." This was the only medicine he took, and in a few days he was able to walk about his room.

In a third caſe, I tried the acid without the opium, but it did not ſucceed; I then united them, and it effected immediate relief.

I was ſtill unwilling to perſuade myſelf into the belief of its being a ſpecific remedy, until a caſe of ſo extraordinary a nature occurred, as compelled me to decide, unequivocally, in its favour. A young lad, ſixteen years of age, fell over a dredging-boat into the water, in the month of July laſt: indiſpoſition ſucceeded; but to what degree I could not determine, as another practitioner was at firſt called in to his aſſiſtance. Finding no relief, they applied to me, requeſting ing my attendance; this was near a month after the accident. The remedies I prefcribed, failed in their efficacy; his friends, defpairing of relief, requefted me not to trouble myfelf to attend him any more, (they lived fix miles from me) faying, he muft be left to his fate, being affured a day or two more would finifh the fcene. At this time, I recommended the acid and anodyne; and with great difficulty it was, that they could be perfuaded to give it a trial; twenty-four hours had not elapfed before he began to find relief, fo that before the expiration of the fourth day, he left off the remedy. The difeafe returned; re-application to the drops again removed it; and in a very fhort fpace of time, without any other medicine intervening, he became as hearty as ever he had been. The manifeft advantage of the medicine was recognized by the parents; nor did they fpare unjuft reflections that I had not employed it before.

I have fince mentioned thefe circumftances among a circle of my medical friends, only one of whom has had an opportunity of giving it a trial, and joins heartily with me in refpect to its falutary effect.

In one cafe the ext. pap. alb. was made ufe of with equal advantage, but in larger dofes, fo as to confider it but of half the ftrength of the opium.

Hoping this general relation of circumftances may be fufficient to induce gentlemen, efpecially in the army and navy, to give it a fair and impartial trial, and to make fuch reports thereof, as may ftill further the knowledge of the remedy, fhould it in future fucceed, it will adminifter fome confolation to my mind; and that I may reflect my life has not been fpent in vain, is one of the higheft aims of my ambition.

